# Research trends and hotspots of post-stroke upper limb dysfunction: a bibliometric and visualization analysis

**DOI:** 10.3389/fneur.2024.1449729

**Published:** 2024-10-02

**Authors:** Qingqing Tang, Xinyue Yang, Mengmeng Sun, Min He, Ren Sa, Kaiqiang Zhang, Bing Zhu, Tie Li

**Affiliations:** ^1^Department of Acupuncture and Tuina, Changchun University of Chinese Medicine, Changchun, China; ^2^School of Medicine, Lishui University, Lishui, China; ^3^Northeast Asia Research Institute of Traditional Chinese Medicine, Changchun University of Chinese Medicine, Changchun, China; ^4^Department of Traditional Chinese Medicine and Acupuncture, Sanya Traditional Chinese Medicine Hospital, Sanya, China; ^5^Institute of Acupuncture and Moxibustion, China Academy of Chinese Medical Sciences, Beijing, China

**Keywords:** bibliometric, literature review, stroke, upper limb dysfunction, visualized analysis

## Abstract

**Background:**

The global prevalence of stroke has been increasing. Motor dysfunction is observed in approximately 55 to 75% of stroke patients, with upper limb impairment affecting around 85% of them. Following upper limb dysfunction, the body’s recovery time is not only slower compared to the lower limbs, but the restoration of its fine motor skills is significantly more challenging, greatly impacting the daily lives of patients. Consequently, there is an increasing urgency for study on the upper limb function in stroke.

**Methods:**

A search was conducted in the Web of Science Core Collection: Science Citation Index Expanded (SCI-Expanded) database for material published from January 1, 2004 to December 31, 2023. We included all relevant literature reports and conducted an analysis of annual publications, countries/regions, institutions, journals, co-cited references, and keywords using the software packages CiteSpace, VOSviewer, and Bibliometrix R. Next, we succinctly outlined the research trends and hotspots in post-stroke upper limb dysfunction.

**Results:**

This analysis comprised 1,938 articles from 1,897 institutions, 354 journals, and 53 countries or regions. A yearly rise in the production of publications was noted. The United States is the foremost nation on the issue. Northwestern University has the most amounts of papers compared to all other institutions. The journal Neurorehabilitation and Neural Repair is a highly significant publication in this field, with Catherine E. Lang serving as the principal author. The majority of the most-cited references focus on subjects such as the reliability and validity of assessment instruments, RCT of therapies, systematic reviews, and meta-analyses. The intervention measures primarily comprise three types of high-frequency phrases that are related, as determined by keyword analysis: intelligent rehabilitation, physical factor therapy, and occupational therapy. Current areas of focus in research include randomized clinical trials, neurorehabilitation, and robot-assisted therapy.

**Conclusion:**

Current research has shown a growing interest in studying upper limb function assessment, occupational therapy, physical therapy, robot-assisted therapy, virtual reality, brain-computer interface, telerehabilitation, cortical reorganisation, and neural plasticity. These topics have become popular and are expected to be the focus of future research.

## 1 Introduction

According to 2019 Global Burden of Disease Study research, stroke is still the second largest cause of mortality (11.6% of total deaths) and the third major cause of disability (1). Motor dysfunction is a common sequela in patients after recovery from stroke, accounting for 50–70% of all sequelae (2). Studies have shown that about 80% of patients with acute stroke and more than 55% of patients with chronic stroke have upper limb motor dysfunction (3, 4). About 30% of patients find it difficult to control the fine movement of the upper limbs (5). The main causes of upper limb motor dysfunction after stroke are muscle weakness, and contracture, changes in muscle tone, joint subluxation and impaired motor control, which in turn affect the coordination and execution of arms, palms and fingers, resulting in difficulty in feeding and dressing themselves and other daily activities, thus directly reducing the quality of life of stroke patients and bringing a huge burden to families and society (6). Improving upper limb motor dysfunction is closely related to patient daily lives and is an important priority for stroke rehabilitation (7).

The extensive projection of upper limb nerve fibers in the cerebral cortex results in a relatively large proportion of nerve innervation. Consequently, functional damage is more pronounced, necessitating a longer recovery time for the upper limb. Additionally, the rehabilitation training for recovery is more challenging (8). Researchers have dedicated themselves to the study of post-stroke upper limb dysfunction, exploring various interventions such as physical therapy, psychotherapy, robotic rehabilitation, and other treatments. These approaches have been progressively integrated into the rehabilitation of upper limb function following a stroke, and several clinical studies have been conducted to confirm their efficacy (4, 9–12). At the same time, the diagnosis, prevention, screening and efficacy evaluation tools for upper limb dysfunction after stroke are also constantly improving (13–16). On this basis, scientists have also carried out a series of studies to explore the mechanism of post-stroke upper limb dysfunction (17–20). Over the past 20 years, a large amount of literature related to post-stroke upper limb disorders have been published, but the research directions and hotspots in this field have not been clearly summarized. It is necessary to conduct a bibliographic analysis of research on motor function rehabilitation after stroke to fully utilize the existing research foundation and identify the research hotspots, which is helpful to carry out more in-depth scientific research in this field.

Bibliometrics is an interdisciplinary field that utilizes statistical and quantitative methods to study, evaluate, and analyze scientific literature. It serves to review the current state of specific research areas, identify hotspots, discern trends, and assess the quality of publications. Among the databases used for such analyses, the Web of Science Core Collection (WoSCC) stands out as the predominant choice (21). The application of information visualization technology and methods can intuitively show the development process, research status, research hotspots and development trends of the research field, and help to identify the current development status and emerging research directions (22). However, from the perspective of bibliometrics, the comprehensive knowledge structure, evolutionary pathways and research hotspots in the field of post-stroke upper limb disorders have not been analyzed. Bibliometric articles related to upper limb dysfunction after stroke have not yet been published.

CiteSpace and VOSviewer are the most widely used software tools for literature information visualization. VOSviewer and CiteSpace are visual analysis software developed by Professor Van Eck of Leiden University in the Netherlands and Professor Chen Chaomei of Drexel University in the United States, respectively (23–25). VOSviewer primarily deconstructs the relationships of elements to be analyzed by distance, while CiteSpace focuses on graphics and connections, showing the strength of the relationships among the analyzed elements. The main features of both are their rich graphical presentations and clear displays, making the results of bibliometric analyses easy to interpret. CiteSpace and VOSviewer are employed to visualize bibliometric networks encompassing countries, regions, institutions, authors, journals, citations, keywords, and more. Additionally, R studio with Bibliometrix R package was utilized to preprocess data related to publication trends, country/region analysis, and cited analysis.

The purpose of this study is to comprehensively review the literature published in the past 20 years on the diagnosis, treatment, functional evaluation and mechanism research of upper limb function after stroke, explore the development status, research hotspots and development trends in this field, and identify emerging research directions. By using bibliometric study and visualization analysis, it provides researchers with visual information and potential research directions in a clear and intuitive way. Overall, this study provides a brief overview of the important progress, trends and hotspots of upper limb injury after stroke in the past two decades, and provides guidance for future research in the field of upper limb function after stroke.

## 2 Materials and methods

### 2.1 Search strategy

This study specifically examines the upper limb dysfunction following a stroke. When developing our search tactics, we carefully considered all potential alternate descriptions of post-stroke upper limb dysfunction. We broadened the search scope to ensure the retrieval of literature containing diverse descriptions of post-stroke upper limb dysfunction. The precise search methodologies are documented in Supplementary material S1. The studies were obtained from the SCI-expanded database of the Web of Science Core Collection. In light of the significant advancements in post-stroke upper limb dysfunction, we have selected a specific timeframe of 20 years, starting on January 1, 2004, and ending on December 31, 2023, for our analysis. The scope of publications was restricted to articles and reviews.

### 2.2 Inclusion criteria

Publications that encompassed people or animals with a verified diagnosis of post-stroke upper limb dysfunction, as well as publications where patients or animals were diagnosed with stroke and had upper limb function impairment, were incorporated. Furthermore, the research should primarily address the study of epidemiology, encompassing demographic characteristics and risk factors. It should also explore the processes, pathophysiology, diagnosis, screening, prevention, management, and therapy of upper limb disability following a stroke.

### 2.3 Exclusion criteria

Completely irrelevant literature studies and those that focused on post-stroke upper limb dysfunction relevant disease, but the research content only focused on the epidemiology (demographic factors and risk factors), mechanisms, diagnosis, pathophysiology, screening, prevention, management, or treatment of other relevant diseases but not post-stroke upper limb dysfunction were excluded.

**Figure 1:**
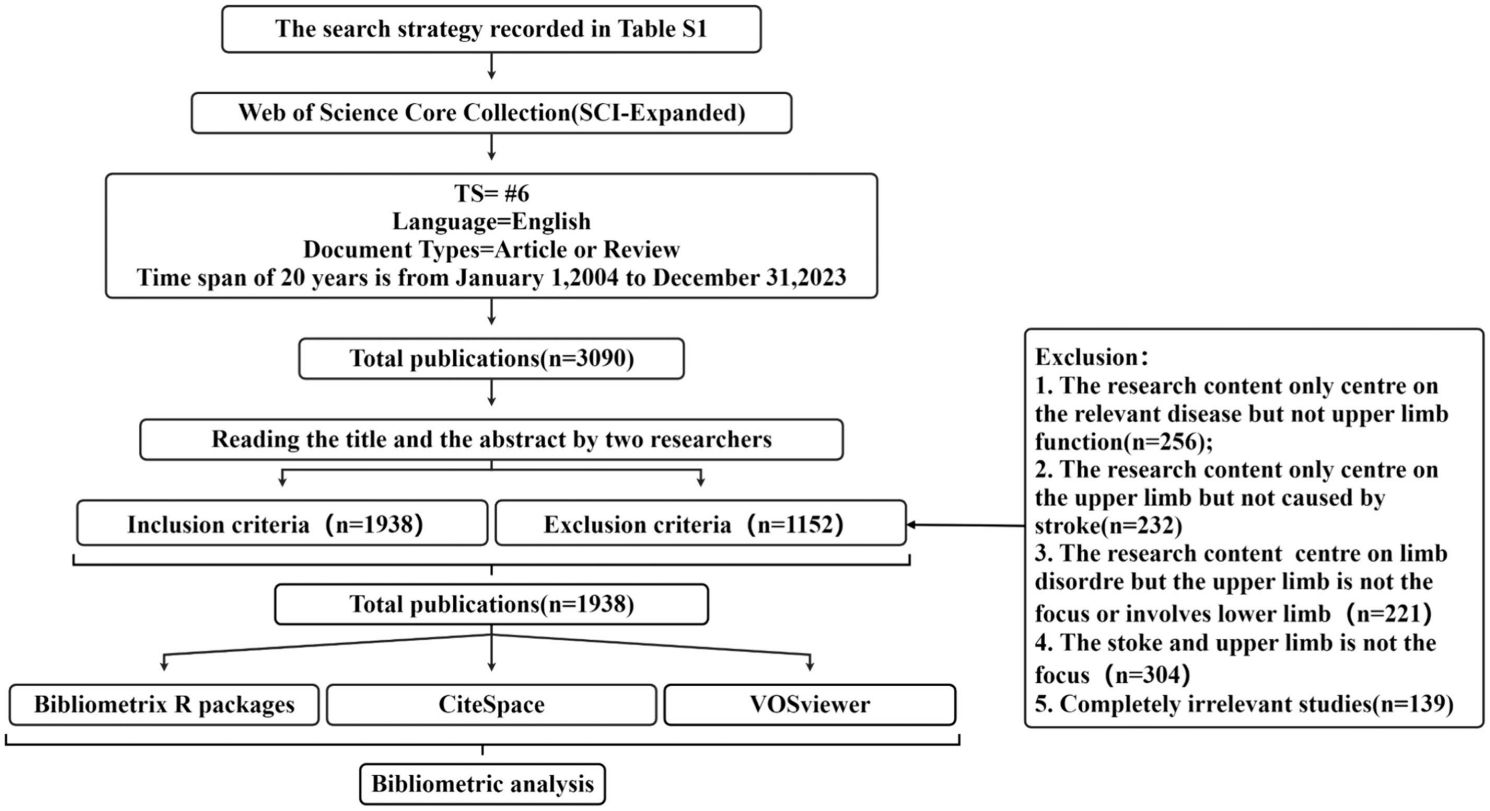
Flow chart of literature selection.

Two researchers (QT and KZ) independently screened the retrieved literature on stroke research by reviewing the titles and abstracts in accordance with the established inclusion and exclusion criteria. Their agreement indicated a significant level of concordance. Any discrepancies that arose during this process were discussed and resolved with the involvement of the third author (XY). Figure 1 depicts the screening process for publications, and the reasons for exclusion are recorded in Supplementary material S2.

### 2.4 Analytical tool

The relevant literature meeting the inclusion criteria was exported as a plain text file of “full record and cited references” and named “stroke.txt,” and visual analysis was performed using the R studio with Bibliometrix R package (4.3.2), Microsoft Excel 2019, CiteSpace 6.2.R6 (Drexel University, PA, United States), and VOSviewer 1.6.2 (Leiden University, Netherlands) with Pajek.

## 3 Results

### 3.1 Publication trends

**Figure 2:**
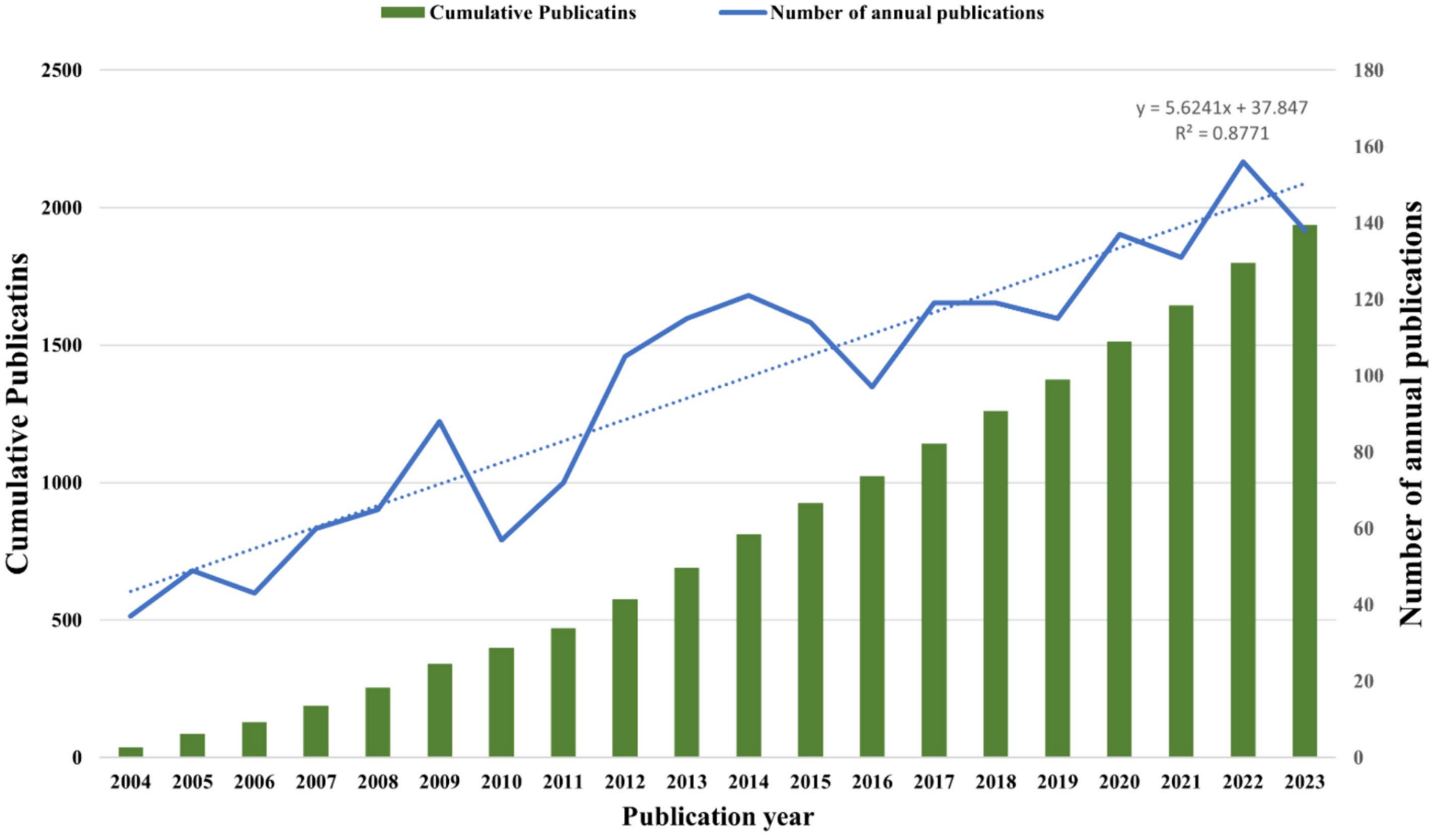
Annual trends of global publications over the past 20 years. The green column indicates the cumulative number of documents, while the blue solid line represents the annual number of documents published. The blue dotted line illustrates the trend line of publication (*R*^2^), reflecting the publication trends in this field over the past 20 years.

According to our search strategy, from 2004 to the end of 2023, a total of 1,938 publications met our inclusion criteria, consisting of 1,800 (92.88%) articles and 138 (7.12%) reviews, then we exported these documents as “stroke.txt” files and imported to the Bibliometrix R package to statistical analysis. We exported the complete information of these 1938 articles in excel format in Supplementary material S3. Then based on the excel file of annual scientific output exported by Bibliometrix, we used Excel 2019 to generate the annual trends chart of post-stroke upper limb dysfunction in the past 20 years. Figure 2 shows that since 2004, the number of publications has shown an overall upward trend. The number of annual publications increased rapidly and peaked in 2022 (*n* = 156). Overall, the amount of knowledge in the field of upper limb dysfunction after stroke has shown a linear growth trend (*R*^2^ = 0.8711), which highlights the increasing attention of researchers in recent years and reflects the increasing interest in this field.

### 3.2 Country/region analysis

**Figure 3:**
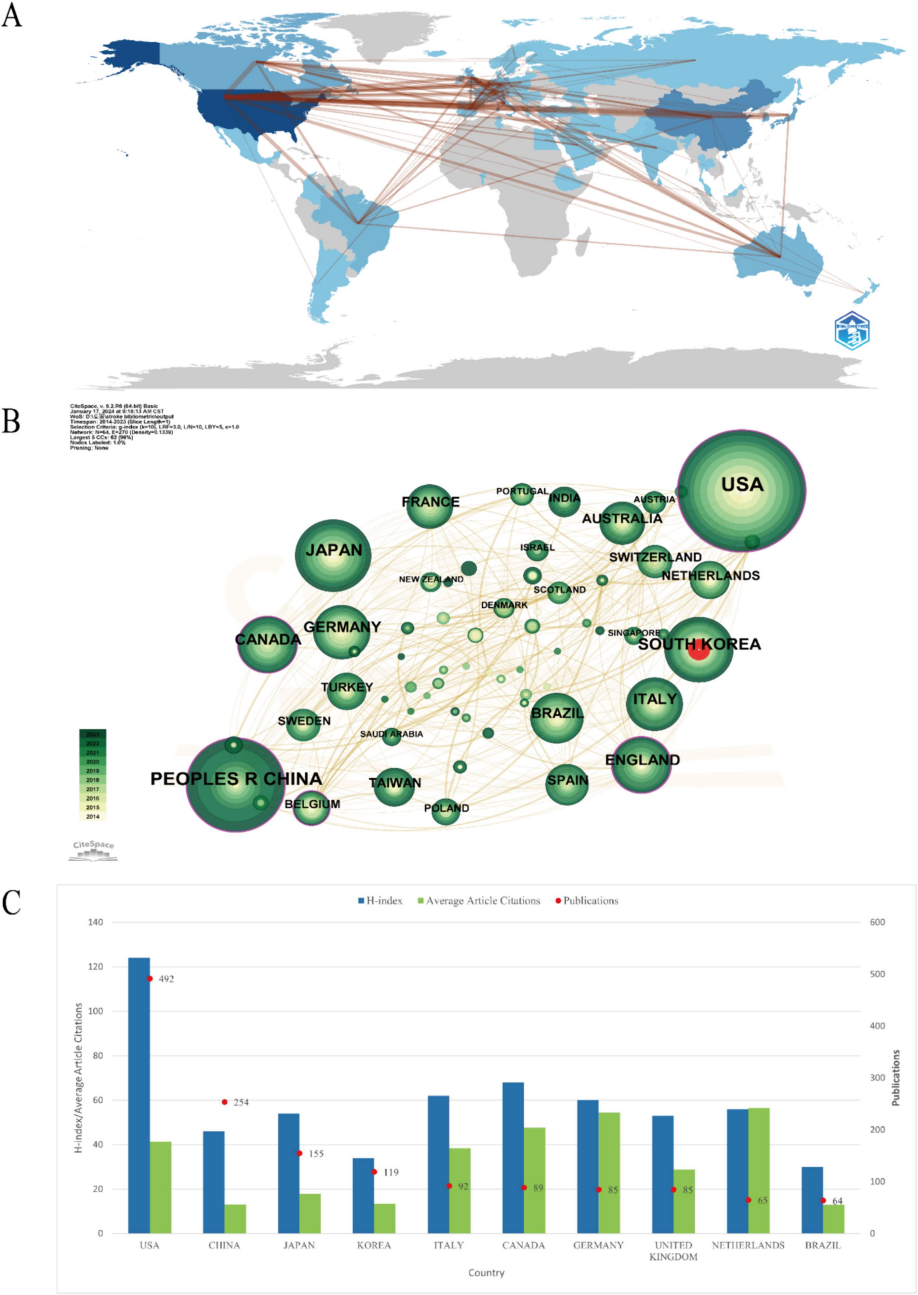
Geographical distribution and network of countries and regions in post-stroke upper limb function. **(A)** Network of international cooperation. The size of the nodes represents the number of articles. The thickness of the curves represents the strength of collaboration. **(B)** Co-occurrence network of countries and regions. **(C)** Number of publications in the top 10 countries and regions and their *H*-index, average article citations, and publications.

A total of 53 countries and regions have published articles in this field. When ranked by the number of publications, the United States ranked first with 492 publications, accounting for more than a quarter of the total number of publications, and is the core research country in this field, followed China (*n* = 254, 13.11%) and Japan (*n* = 155, 8%). Eight other countries (33.5% of the total) published more than 50 publications. Overall, Americas published the largest number of post-stroke upper limb related studies, followed by Asia and Europe. A map of the contributions of countries or regions in this field is available from Bibliometrix, as shown in Figure 3A. The field has formed a wide range of research cooperation networks between the United States, Europe, Asia and Oceania around the world. By visualizing the network of cooperation between countries, cooperation between countries or regions can be shown.

The CiteSpace software was utilized with the following parameter settings: the time span is from January 2004 to December 2023, and the years per slice is 1. The node type is set to country, and set the scale factor *k* = 9 for load all the nodes. Figure 3B shows the major cooperation networks of countries, with nodes with purple rings representing high centrality (centrality ≥0.1), indicating that they are considered to be of high importance and influence (11). The United States (*c* = 0.31), the United Kingdom (*c* = 0.23), China (*c* = 0.2), Belgium (*c* = 0.2) and Canada (*c* = 0.14) showed higher centrality in this field. The nodes with red rings indicate that the annual citation frequency increases or increases rapidly and continuously. South Korea shows the strongest citation burst in a certain period of time, indicating that the academic activity is high and its research has received extensive attention from scholars.

The national *H*-index serves as an indicator for assessing the quality of a country’s scientific research output within a specific field. A higher *H*-index signifies greater quality and influence of the country’s scientific research contributions in that area (26). We used R studio to extract the country’s *H*-index. By analyzing the *H*-index in related fields, we find that the United States (*H* = 124) has the highest score and the greatest overall scientific research influence, followed by Canada (*H* = 68), Italy (*H* = 62) and Germany (*H* = 60). Then we obtained the number of publications and average article citations from Bibliometrix. Figure 3C shows the top 10 countries in the number of publications on upper limb dysfunction after stroke with their corresponding *H*-index values and citations. Among them, American publications were still the most frequently cited publications, which were cited 20,308 times. Germany (ac = 4,622) ranked second, and it also had the highest average article citation rate (aac = 54.4).

### 3.3 Institutional analysis

The VOSviewer software was utilized with the following parameter settings after import the “stroke.txt”: type of analysis was chosen co-authorship, unit of analysis was chosen organizations, counting method was chosen full counting, and the minimum thresholds for documents, citations of an organization were established at 5 and 0, respectively. Combined with Pajek software to adjust the node to generate network diagram.

**Figure 4:**
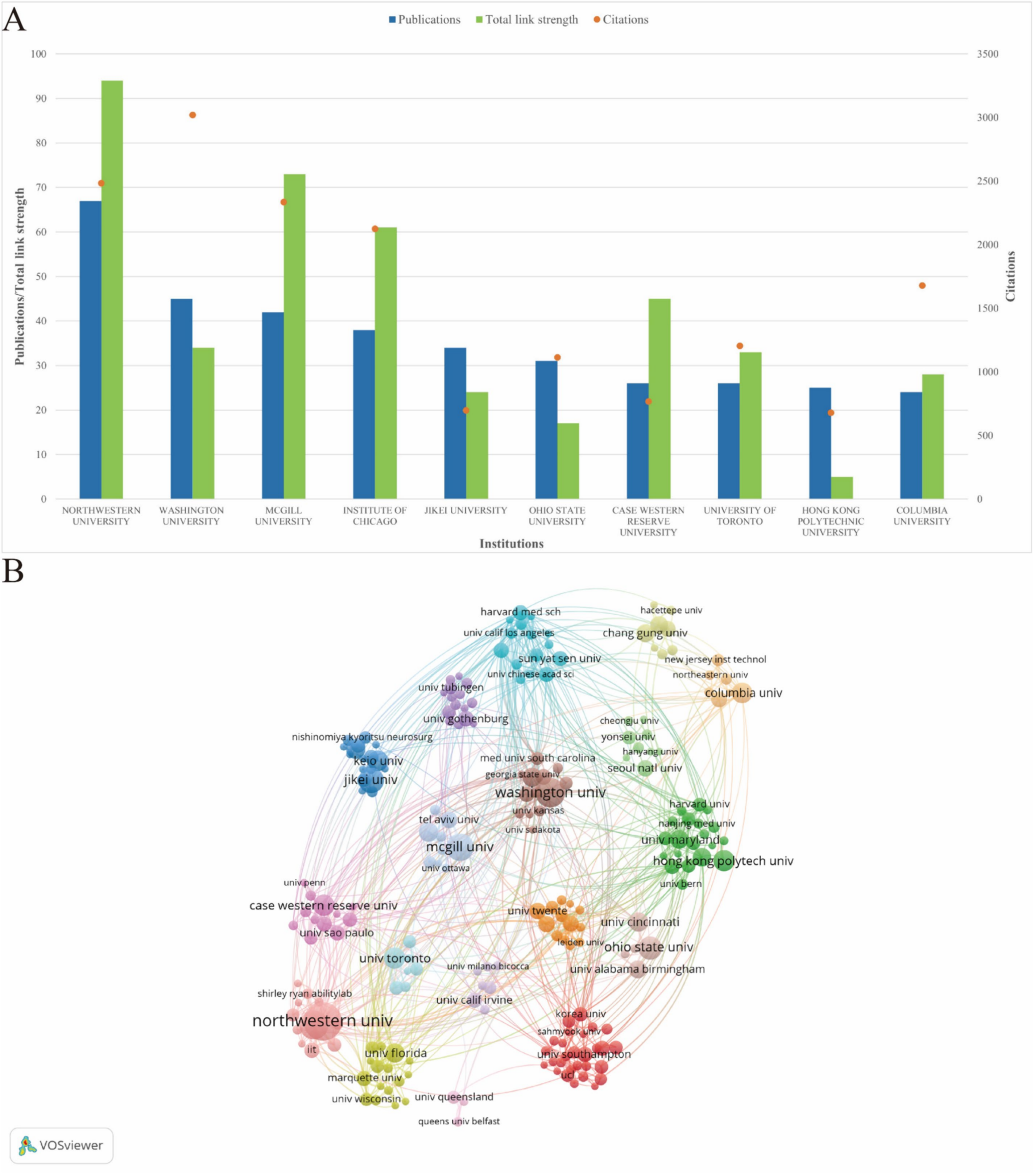
Information and network of institutions in post-stroke upper limb function. **(A)** Number of publications of the top 10 institutions (total link strength indicates the cooperative activity of the institution). **(B)** Network of institutions engaged in post-stroke upper limb function. The node size represents the number of articles; the curve thickness represents the strength of collaboration; the different collaboration groups are sorted by color.

From 2004 to the end of 2023, a total of 1,897 institutions participated in the publication of papers related to upper limb dysfunction after stroke. Figure 4A shows the top 10 institutions with the largest number of publications. Northwestern University (*n* = 67, 3.46%), Washington University (*n* = 45, 2.32%), and McGill University (*n* = 42, 2.17%) are the top three countries with the largest number of publications and citations in the world, showing they have wide interest and strong influence in this field. In terms of national collaboration, Figure 4B shows that the cooperation of various institutions is active. Northwestern University, Washington University, McGill University, the Institute of Chicago, Jikei University, Ohio State University, Case Western Reserve University, the University of Toronto, Hong Kong Polytechnic University, Columbia University, and Keio University were at the center of such collaborations.

### 3.4 Authors and cited authors

A total of 7,693 authors were associated with publications related to the study of upper limb after stroke. The VOSviewer software was utilized with the following parameter settings: type of analysis was chosen co-authorship, unit of analysis was chosen authors, counting method was chosen full counting, and the minimum thresholds for documents, citations of an author were established at 5 and 0, respectively.

**Figure 5:**
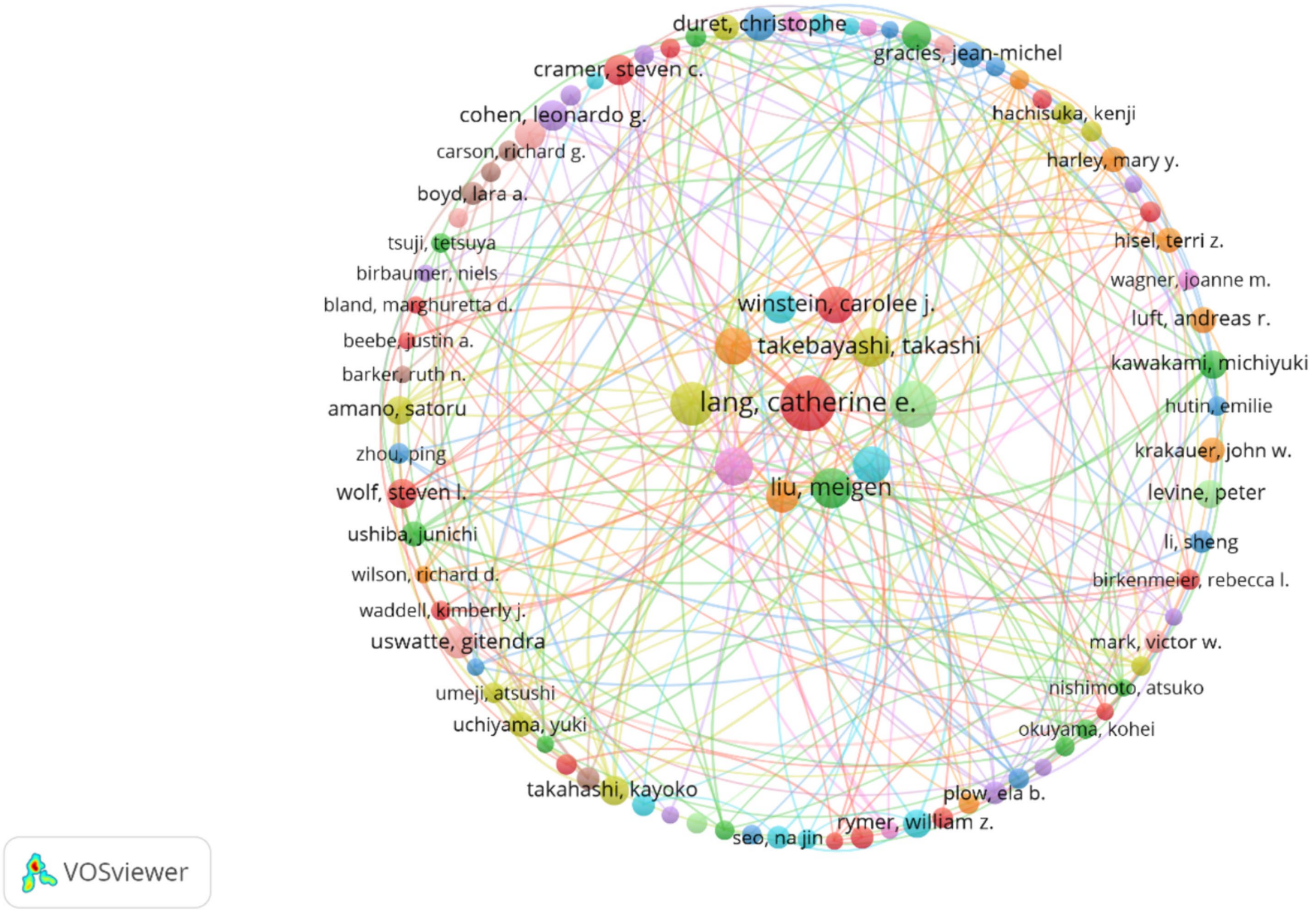
Network of authors engaged in post-stroke upper limb dysfunction. Nodes represent authors (the larger the circle, the greater the number of publications). The lines between nodes represent the cooperation between two authors of the same article (the wider the line, the more frequent the cooperation).

Through the analysis of the authors of the literature, we can determine the representative scholars and core research forces in this field. As shown in Table 1, Catherine E. Lang is the author with the largest number of published articles and citations (*n* = 34, 1.75%). Masahiro Abo and Kazuhisa Domen showed higher link strength and closer cooperation with other authors. The local *H*-index refers to *h* publications in a field where each paper is cited at least *h* times. It is an important indicator to comprehensively measure the quantity and quality of researchers’ output. Catherine E. Lang (*h* = 25), Stephen J. Page (*h* = 21), and Mindy F. Levin (*h* = 20) are the most influential researchers in this field, with high-quality publications. In terms of cooperation, combined with Pajek software to adjust the node to generate Figure 5 shows network cooperation with Catherine E. Lang, Takashi Takebayashi, and Meigen Liu as the core.

**Table 1:** Ranking of top-10 most productive authors in recent 20 years.

Rank	Author	Documents	Citations	Total link strength	*H*-index
1	Lang C. E.	34	2,310	71	25
2	Abo M.	33	661	82	15
3	Page S. J.	26	1,447	17	21
4	Levin M. F.	24	797	35	20
5	Domen K.	23	330	81	11
6	Liu M.	20	707	64	13
7	Kwakkel G.	19	2,052	12	16
8	Kakuda W.	19	565	61	15
9	Takebayashi T.	19	209	74	8
10	Dromerick A. W.	18	1,179	45	17

### 3.5 Journal analysis

**Figure 6:**
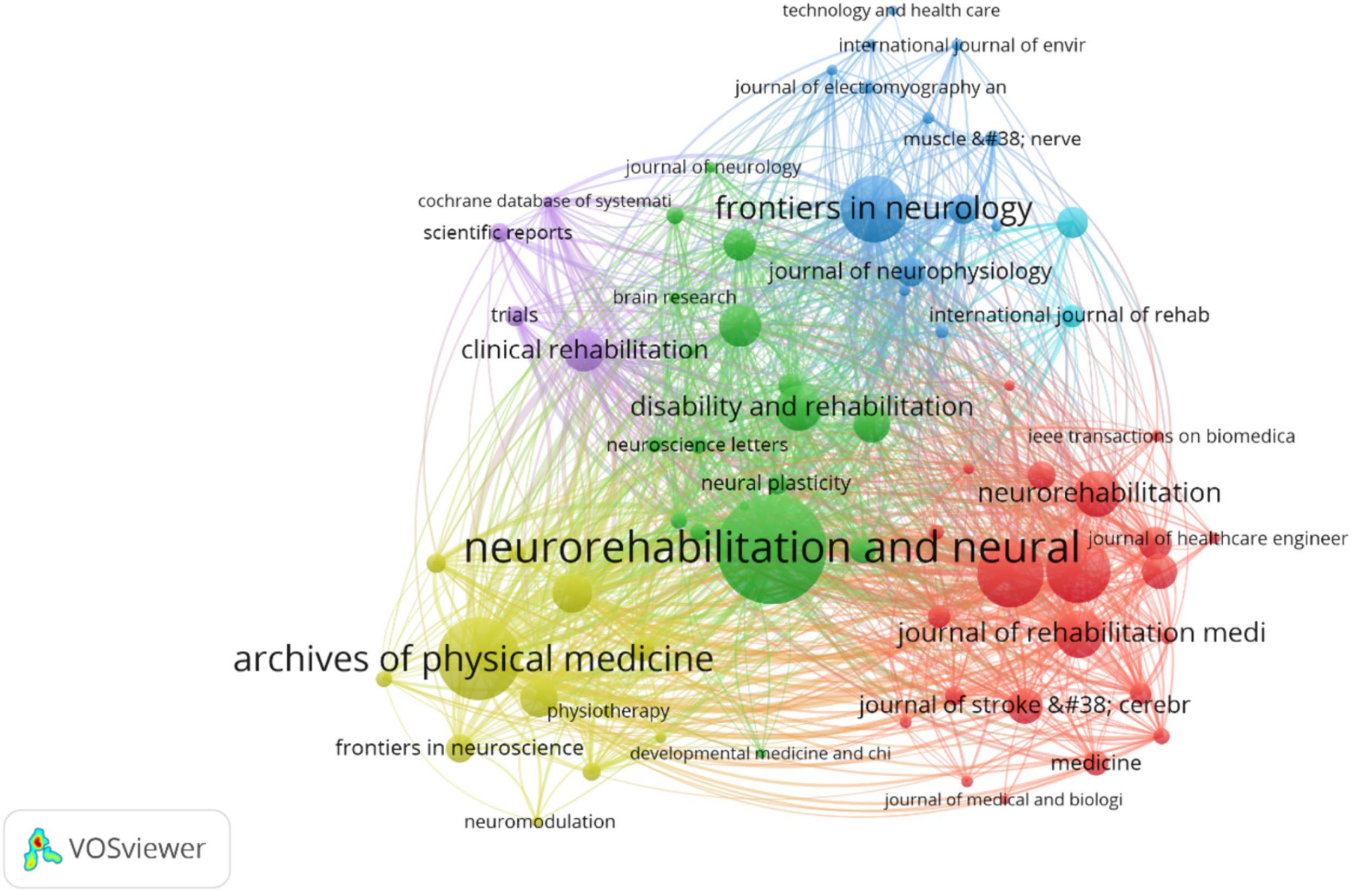
Network of journals engaged in in post-stroke upper limb dysfunction.

From 2004 to the end of 2023, researchers published studies on upper limb dysfunction after stroke in 354 academic journals. Figure 6 is the network diagram between them generate by VOSviewer software with parameter settings that type of analysis was chosen citation and unit of analysis was chosen sources. Neurorehabilitation and Neural Repair ranked first with 154 articles (8.95%), followed by Archives of Physical Medicine and Rehabilitation (*n* = 103, 5.31%) and Frontiers in Neurology (*n* = 75, 3.87%). Journals with an *H*-index greater than 30 are considered to be high-impact journals in this field. Journals on *H*-index 30 include Neurorehabilitation and Neural Repair (*h* = 57), Archives of Physical Medicine and Rehabilitation (*h* = 40), Journal of NeuroEngineering and Rehabilitation (*h* = 30), and Stroke (*h* = 30).

### 3.6 Cited analysis and document type quantification

The co-citation analysis of references reflects the structure and dynamics of the knowledge field and also reveals the research frontier and knowledge base. Export “Most Local Cited Documents and “Most Local Cited References” data from Bibliometrix. Table 2 lists the 10 most frequently cited articles among the 1,938 articles retrieved. “Reliability and validity of arm function assessment with standardized guidelines for the Fugl-Meyer Test, Action Research Arm Test, and Box and Block Test: a multicentre study” is the most cited publication. In addition, we exported the data from Bibliometrix of references cited in the included literatures and ranked them according to the frequency of citations. And a total of 33,884 references were cited in 1,938 publications, of which the most cited reference was “Interrater reliability of a modified Ashworth scale of muscle spasticity,” and the top 10 cited articles are listed in Table 3, they are the most influential literature in this field. It includes three articles published in JAMA, Stroke and The Lancet Neurology in the past 20 years, which are clinical experimental articles, randomized controlled experimental articles and review articles, they are the influential articles in this field in the past 20 years.

**Table 2:** Top 10 publications in post-stroke upper limb function.

Rank	Title	Author	Source	Citation	Year	DOI
1	Reliability and validity of arm function assessment with standardized guidelines for the Fugl-Meyer Test, Action Research Arm Test and Box and Block Test: a multicentre study	Platz T.	Clinical Rehabilitation	134	2005	10.1191/0269215505cr832oa
2	Effects of robot-assisted therapy on upper limb recovery after stroke: a systematic review	Kwakkel G.	Neurorehabilitation and Neural Repair	98	2008	10.1177/1545968307305457
3	Clinically important differences for the upper-extremity Fugl-Meyer scale in people with minimal to moderate impairment due to chronic stroke	Page S. J.	Physical Therapy	95	2012	10.2522/ptj.20110009
4	Reliability and validity of the upper-extremity Motor Activity Log-14 for measuring real-world arm use	Uswatte G.	Stroke	81	2005	10.1161/01.STR.0000185928.90848.2e
5	Interventions for improving upper limb function after stroke	Pollock A.	Cochrane Database of Systematic Reviews	72	2014	10.1002/14651858.CD010820.pub2
6	The Motor Activity Log-28: assessing daily use of the hemiparetic arm after stroke	Uswatte G.	Neurology	64	2006	10.1212/01.wnl.0000238164.90657.c2
7	Estimating minimal clinically important differences of upper-extremity measures early after stroke	Lang C. E.	Archives of Physical Medicine and Rehabilitation	59	2008	10.1016/j.apmr.2008.02.022
8	Task-specific training with trunk restraint on arm recovery in stroke: randomized control trial	Michaelsen S. M.	Stroke	54	2006	10.1161/01.STR.0000196940.20446.c9
9	Rehabilitation of motor function after stroke: a multiple systematic review focused on techniques to stimulate upper extremity recovery	Hatem S. M.	Physical Therapy Frontiers in Human Neuroscience	53	2016	10.3389/fnhum.2016.00442
10	Mirror therapy improves hand function in subacute stroke: a randomized controlled trial	Yavuzer G.	Archives of Physical Medicine and Rehabilitation	49	2008	10.1016/j.apmr.2007.08.162

**Table 3:** Top 10 cited references in post-stroke upper limb function.

Rank	Title	Author	Source	Citation	Year	DOI
1	Interrater reliability of a modified Ashworth scale of muscle spasticity	Bohannon R. W.	Physical Therapy	291	1987	10.1093/PTJ/67.2.206
2	Effect of constraint-induced movement therapy on upper extremity function 3 to 9 months after stroke: the EXCITE randomized clinical trial	Wolf S. L.	JAMA	245	2006	10.1001/JAMA.296.17.2095
3	The Fugl-Meyer assessment of motor recovery after stroke: a critical review of its measurement properties	Gladstone D. J.	Neurorehabilitation and Neural Repair	224	2002	10.1177/154596802401105171
4	Probability of regaining dexterity in the flaccid upper limb: impact of severity of paresis and time since onset in acute stroke	Kwakkel G.	Stroke	185	2003	10.1161/01.STR.0000087172.16305.CD
5	Assessing Wolf motor function test as outcome measure for research in patients after stroke	Wolf S. L.	Stroke	171	2001	10.1161/01.STR.32.7.1635
6	A performance test for assessment of upper limb function in physical rehabilitation treatment and research	Lyle R. C.	International Journal of Rehabilitation Research	148	1981	10.1097/00004356-198112000-00001
7	Recovery of upper extremity function in stroke patients: the Copenhagen Stroke Study	Nakayama H.	Archives of Physical Medicine and Rehabilitation	144	1994	10.1016/0003-9993(94)90161-9
8	Reliability and validity of arm function assessment with standardized guidelines for the Fugl-Meyer Test, Action Research Arm Test and Box and Block Test: a multicentre study	Platz T.	Clinical Rehabilitation	134	2005	10.1191/0269215505CR832OA
9	Reliability of the Fugl-Meyer assessment of sensorimotor recovery following cerebrovascular accident	Duncan P. W.	Physical Therapy	125	1983	10.1093/PTJ/63.10.1606
10	Motor recovery after stroke: a systematic review	Langhorne P.	The Lancet Neurology	123	2009	10.1016/S1474-4422(09)70150-4

A total of 33,884 references were cited in 1,938 articles, which were ranked according to citation frequency.

**Figure 7:**
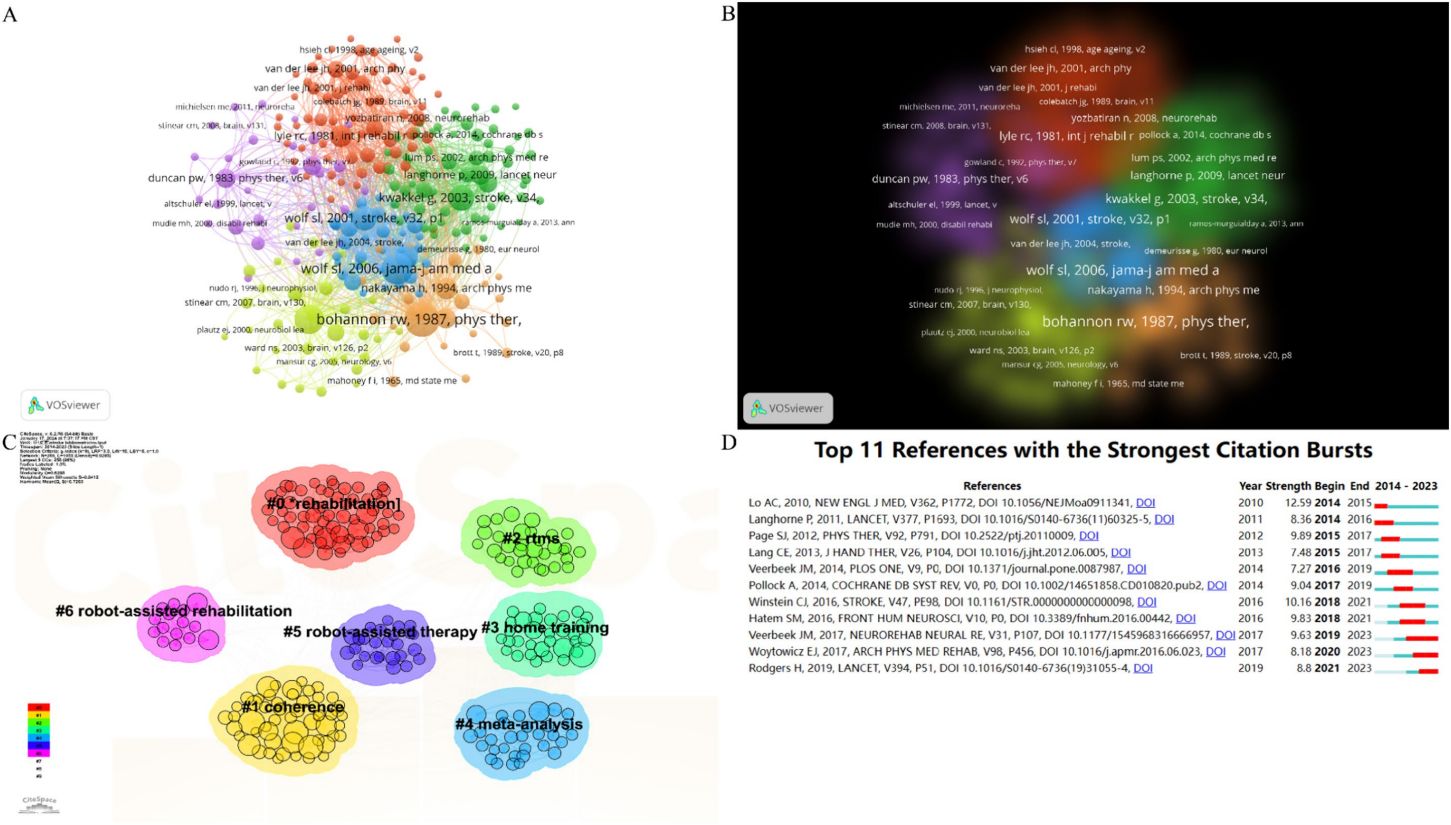
Local cited documents visualization. **(A)** Network visualization of references. The size of the nodes represents the number of occurrences; the thickness of the curve represents the strength of collaboration; the different colors represent the different clusters. **(B)** Density visualization of references. **(C)** Cluster diagrams of publications. **(D)** The strongest citation bursts of the top 11 publications.

At least two articles are referred to at the same time in at least one report, indicating coreference between them. The VOSviewer software was utilized with the following parameter settings: type of analysis was chosen co-citation, unit of analysis was chosen cited references. Figure 7A presents the network visualization map with six clusters in different colors, the size of nodes is proportional to the coreference times of publications, the thicker the lines between two nodes, the higher the frequency of their coreference. Figure 7B presents density visualization of references, different clusters show different colors, with dark colors representing high-density areas and light colors representing low-density areas. Wolf et al. (2006), (1981), Duncan et al. (1983), Kwakkel et al. (2003), Bohannon et al. (1987), and Stinear et al. (2007) were early publications in the field, cited numerous times. These publications could be regarded as the roots of the area.

The CiteSpace software was utilized with the following parameter settings: The time span is from January 2004 to December 2023, and the years per slice is 1. The node type is set to reference, and set the scale factor *k* = 9 for load all the nodes. In the knowledge base map, articles co-cited by another article had a close academic relationship and were therefore grouped into the same cluster. Tags that represent the knowledge base of the cluster were extracted from the titles of the publications. As demonstrated in Figure 7C, 11 crucial tags were extracted, including #rehabilitation, #coherence, #rtms, #home training, #meta-analysis, #robot-assisted therapy, and #robot-assisted rehabilitation.

Burst detection can be utilized to identify references that have received collective attention during a specific period. Chosen burstness, and set the burst *γ* value = 3. The top 11 references with the strongest citation bursts are shown in Figure 7D. Upon analyzing these references, we found that studies authored by Janne M. Veerbeek were widely cited from 2019 to 2023. This is a meta-analysis of randomized controlled trials of robot-assisted therapy for the upper limb after stroke.

**Figure 8:**
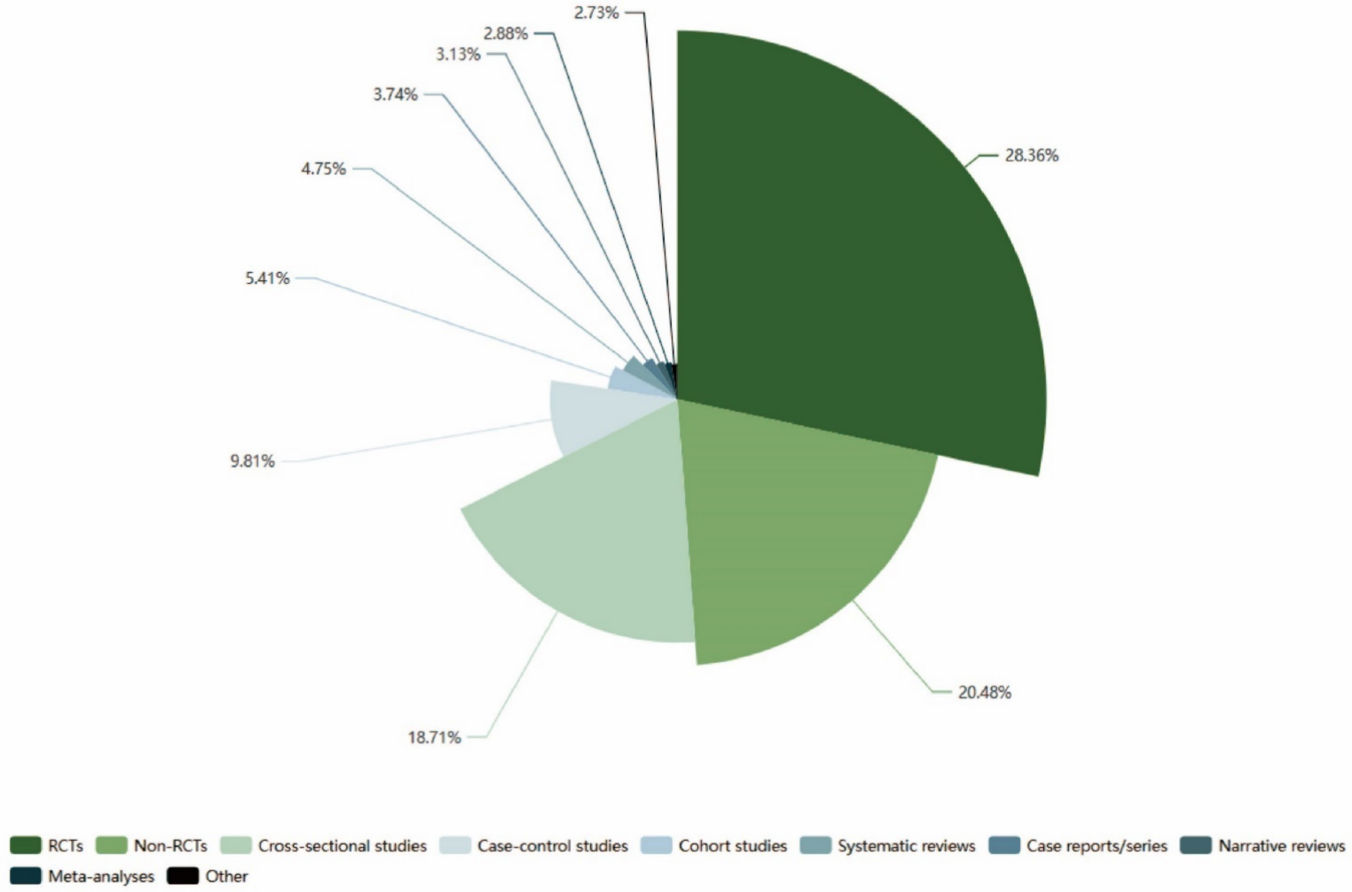
Nightingale rose diagram of document type quantification.

To enhance our understanding of the research design utilized in this field, the literature included in this study was systematically classified and quantified. We categorized all the literature into three main categories: clinical research, secondary research, and other categories. Clinical research is further subdivided into original research and secondary research. Original research encompasses experimental and observational studies. Experimental research is categorized into randomized controlled trials (RCTs) and non-RCTs, while observational studies include case-control studies, cohort studies, cross-sectional studies, and case reports/series. Secondary research comprises narrative reviews, systematic reviews, and meta-analyses. The “other” category includes basic research, interviews, suggestions, comments, and various other types of literature. The results of this classification are presented in Figure 8.

### 3.7 Keywords analysis

**Figure 9:**
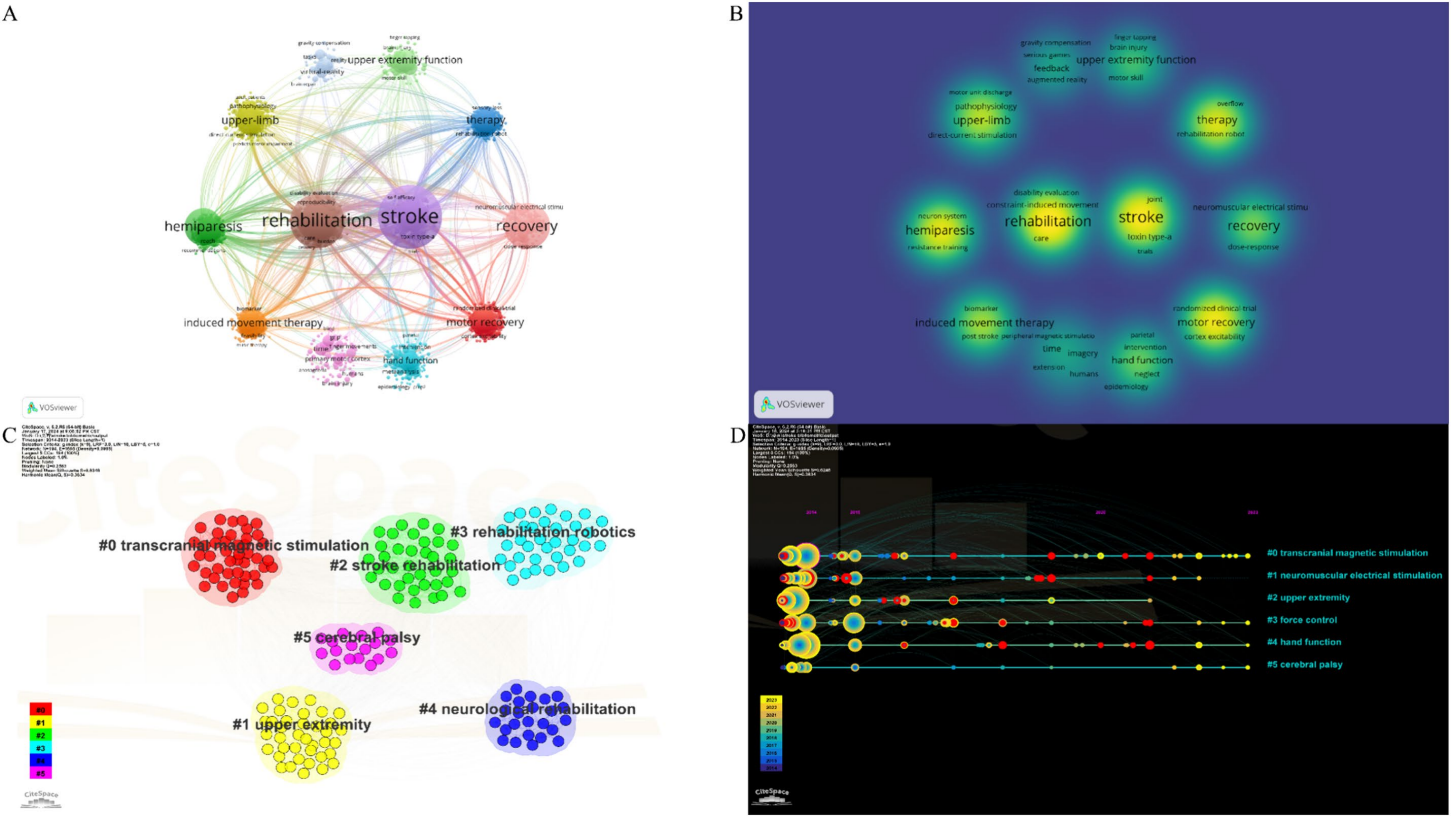
**(A)** The visualization network of co-occurring keywords. **(B)** The density visualization of co-occurring keywords. **(C)** Cluster results of keywords. **(D)** The timeline of keywords.

Keywords are succinct summary of the research content of a paper, and analyzing high-frequency keywords can reveal the main research topics in a given field. The VOSviewer software was utilized type of analysis was chosen co-occurrence and unit of analysis was chosen all keywords, combined with Pajek software to adjust the node to generate network of co-occurring keywords. In the keyword network of co-occurrence analysis of Figure 9A, we selected nodes with occurrences ≥100 or total link strength ≥1,000 from 4,529 keywords in 1,938 articles, and obtained a total of 35 keywords as shown in Table 4. Combined with the density visualization of Figure 9B, we obtained 9 core keywords in the keyword network, including stroke (*n* = 1,222, tls = 14,324), rehabilitation (*n* = 881, tls = 10,516), recovery (*n* = 630, tls = 7,490), hemiparesis (*n* = 415, tls = 5,276), therapy (*n* = 233, tls = 2,750), motor recovery (*n* = 233, tls = 2,750), upper-limb (*n* = 256, tls = 3,151), induced movement therapy (*n* = 217, tls = 2,750), hand function (*n* = 91, tls = 1,149).

**Table 4:** Top 35 keywords in post-stroke upper limb function.

Rank	Keyword	Occurrences	Total link strength	Rank	Keyword	Occurrences	Total link strength
1	stroke	1,222	14,324	19	movement	143	1,809
2	rehabilitation	881	10,516	20	upper limb	144	1,790
3	recovery	630	7,490	21	hand	139	1,736
4	hemiparesis	415	5,276	22	spasticity	133	1,666
5	upper extremity	375	4,579	23	Fugl-Meyer assessment	125	1,609
6	reliability	345	4,253	24	plasticity	117	1,441
7	hemiplegia	294	3,366	25	cortex	111	1,365
8	upper-limb	256	3,151	26	motor function	105	1,284
9	arm	254	3,107	27	paresis	107	1,275
10	motor recovery	229	2,787	28	stroke patients	103	1,227
11	induced movement therapy	217	2,750	29	upper-extremity	96	1,185
12	therapy	233	2,750	30	activation	88	1,153
13	impairment	190	2,339	31	research arm test	91	1,151
14	performance	174	2,156	32	hand function	91	1,149
15	upper extremity function	162	2,047	33	reorganization	90	1,109
16	stroke rehabilitation	159	1,993	34	interrater reliability	88	1,104
17	transcranial magnetic stimulation	156	1,947	35	subacute stroke	78	1,003
18	validity	149	1,824				

**Figure 10:**
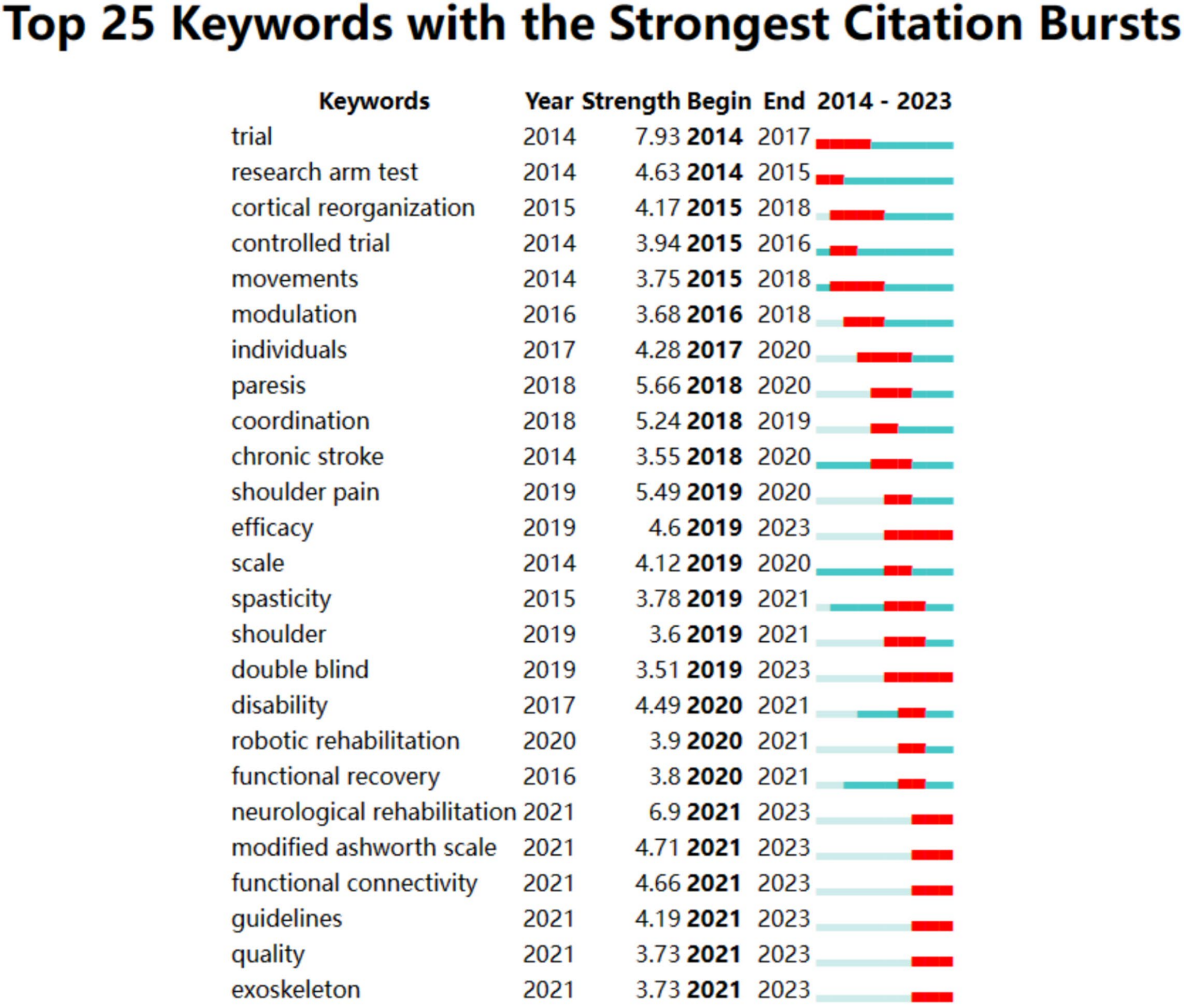
Keywords with the strongest keywords.

The node type of the CiteSpace software is set to keyword and set the scale factor *k* = 9 for load all the nodes. Figure 9C showed the cluster analysis results of keywords including 6 clusters of transcranial magnetic stimulation upper extremity stroke rehabilitation rehabilitation robotics neurological rehabilitation and cerebral palsy. In addition Figure 9D show the visual representation of keyword co-occurrence based on time zone is generated to intuitively understand the trend of each cluster label over time which is helpful to observe the continuous emergence of research trends and the evolution direction of upper limb function rehabilitation research after stroke over time. Chosen burstness and set the burst *γ* value=1.4. Figure 10 shows the top 11 references with the strongest citation bursts in the past two decades. Burst keywords refer to the research hotspots that have been frequently studied during a certain period of time. In recent years burst keywords in this field include neurological rehabilitation (2021–2023) modified Ashworth scale (2021–2023) functional connectivity (2021–2023) guidelines (2021–2023) quality (2021–2023) exoskeleton (2021–2023). These research hotspots not only reflect the current development trends but also provide valuable insights for future research.

## 4 Discussion

With the advent of big data, researchers need to fully understand the development of their research fields. A comprehensive and systematic summary of the research topics, research trends and global research status of upper limb dysfunction after stroke can quickly and provide primarily understanding of the research status of this aspect of stroke. Different from systematic review or meta-analysis, bibliometric analysis comprehensively analyzes the existing literature, intuitively understands research trends, and predicts research hotspots. This study summarizes the research status of upper limb dysfunction after stroke in the past 20 years for the first time through bibliometric analysis.

### 4.1 General information

According to the retrieval strategy, a total of 1,938 articles from 354 academic journals, 1,897 institutions, 53 countries/regions and 1,513 authors in the field of upper limb function after stroke were included in our study. It is worth noting that the total number of publications in this field has increased year by year in the past 20 years, with a peak in 2022, which may be related to the release of the European Neurorehabilitation Upper Limb Clinical Assessment Evidence-based Recommendation (CAULIN) (27). The overall upward trend in the annual number of publications highlights the increasing attention of researchers in recent years, reflecting the increasing interest in research in this field. The publication of a large number of documents is also the basis of our research.

The United States is the core research country in this field, with a large number of publications and scientific research influence; China and Japan rank second in the number of papers published, but through the *H*-index and citation rate of each country, it can be found that China and Japan rank lower, which indicates that their academic influence is limited, and the quality of paper research needs to be improved. Germany, Canada, and Italy are all countries with influence in this field. In general, research in the field of upper limb after stroke is dominant in Europe and the United States, which may be due to the advantages of long-term rehabilitation research practice, rich scientific research resources, advanced technical equipment, a developed academic system, and extensive international cooperation in the field of rehabilitation in Europe and the United States. Northwestern University, Washington University, and McGill University are the three institutions with the largest number of publications in this field, all of which are located in the Americas. Northwestern University is also the most cooperative institution in this field with other institutions and has a core influence in the world. Catherine E. Lang is from the Department of Neurology, Washington University School of Medicine, USA. She is engaged in physical therapy and occupational therapy for upper limb dysfunction after a stroke. She has made outstanding contributions to this field and is the researcher with the largest number of publications and citations. Masahiro Abo, Kazuhisa Domen, Takashi Takebayashi, Stephen J. Page, Mindy F. Levin, and Meigen Liu also have certain influences in this field. Neurorehabilitation and Neural Repair and the Archives of Physical Medicine and Rehabilitation are two core journals in the field of upper limb dysfunction after stroke. They have formed a wide cooperation network with Frontiers in Neurology and the Journal of Neuro Engineering and Rehabilitation. Future scholars should pay attention to these authors and journals in order to quickly access the latest international information and research advances in post-stroke upper limb function.

### 4.2 Research hotspots and trends

In the citation analysis, we summarized the top 10 articles in the field of upper limb function after stroke from 1,938 articles published over the past 20 years, along with the top 10 articles among 33,884 references. Our findings indicate that the reliability and validity of upper limb function assessment tools, such as the Fugl-Meyer Assessment (FMA), Modified Ashworth Scale (MAS), and Motor Activity Log (MAL), have been well established. Furthermore, the conduct of randomized clinical trials related to transcranial magnetic stimulation (TMS), constraint-induced movement therapy (CIMT), robot-assisted therapy, mirror therapy (MT), virtual reality (VR), and other related technologies, as well as systematic evaluations and meta-analyses of various rehabilitation techniques, represent the primary research hotspots in this field. Then we analyzed the cluster results of the publications and the top 11 references with the strongest citation bursts, found that robot-assisted therapy, Fugl-Meyer scale and meta-analysis were the three most popular keywords in this field. In summary, the verification of the reliability and validity of the upper limb function assessment tool based on the Fugl-Meyer scale, the randomized clinical trials based on robot-assisted therapy, and the meta-analysis of various rehabilitation techniques are the research hotspots and trends in this field. RCTs serve as the primary research design method in this area.

Through keyword co-occurrence analysis, we identified a series of high-frequency terms closely related to the topics of interventions, assessment tools, and mechanism research. These terms include “reliability,” “induced movement therapy,” “performance,” “transcranial magnetic stimulation,” “validity,” “spasticity,” “Fugl-Meyer assessment,” “plasticity,” “cortex,” “activation,” “research arm test,” and “hand function,” among others. Through a further comprehensive analysis of the relevant keywords among a total of 4,529 keywords, we categorized interventions for upper limb rehabilitation following a stroke into three main types: motor function-related learning, physical stimulation therapy, and intelligent rehabilitation. Assessment tools can be classified into two categories: scale tools and intelligent assessment tools. Mechanistic studies encompass neural plasticity, functional connectivity, nerve regeneration, and vascular repair. Based on the burst keywords, we propose that the evaluation, robot-assisted therapy, and mechanistic research focused on nerve repair of upper limb function post-stroke will be hot areas of interest and development in the future.

### 4.3 Summary of major findings

Through the analysis and summary of highly cited literature, as well as high-frequency keywords and clustering results, we identified that the primary focus of research in this field pertains to intervention measures, evaluation tools, and mechanisms related to upper limb dysfunction following a stroke. We then examined the keyword information and summarized the key contents across these three aspects, which include constraint-induced movement therapy (CIMT) (*n* = 34, tls = 419), mirror therapy (MT) (*n* = 57, tls = 669), motor imagery (MI) (*n* = 49, tls = 591), neurorehabilitation (*n* = 74, tls = 962), neurological rehabilitation (*n* = 39, tls = 478), transcranial magnetic stimulation (TMS) (*n* = 156, tls = 1,947), repetitive transcranial magnetic stimulation (rTMS) (*n* = 33, tls = 403), magnetic stimulation (*n* = 14, tls = 185), transcranial direct current stimulation (tDCS) (*n* = 18, tls =211), peripheral magnetic stimulation (PMS) (*n* = 4, tls = 54), neuromuscular electrical stimulation (NMES) (*n* = 25, tls = 296), functional electrical stimulation (FES) (*n* = 30, tls = 377), virtual reality (VR) (*n* = 75, tls = 940), brain-computer interface (BCI) (*n* = 36, tls = 440), exoskeleton (*n* = 34, tls = 407), telerehabilitation (*n* = 21, tls = 262), plasticity (*n* = 117, tls = 1,441), neuroplasticity (*n* = 25, tls = 352), reorganization (*n* = 90, tls = 1,109), cortical reorganization (*n* = 65, tls =855), functional connectivity (*n* = 19, tls =249). Subsequently, we engaged in a further discussion.

#### 4.3.1 Intervention measures of post-stroke upper limb rehabilitation

CIMT, MT, and MI are widely utilized rehabilitation techniques for upper limb recovery following a stroke. CIMT involves restricting the use of the unaffected limb, thereby encouraging movement in the hemiplegic limb and maximizing functional recovery (28). However, the high intensity of CIMT training can lead to fatigue and decreased patient compliance, which has led to the development of various modified CIMT programs (m-CIMT). MT, also referred to as mirror visual feedback (MVF), allows patients to imitate and learn movements by observing the healthy side reflected in a mirror. MI involves patients mentally rehearsing movements they have previously performed, without actual execution or muscle contraction, thereby engaging their subjective initiative. RCTs have been conducted in clinical practice to evaluate the effectiveness of various interventions. Research has demonstrated that exercise training methods such as CIMT, MT, and MI can significantly enhance upper limb function in stroke patients (29–35). Furthermore, one study has indicated that m-CIMT may represent the most effective unconventional intervention for upper limb rehabilitation after a stroke (36).

The rehabilitation treatment of the central and peripheral nervous systems, stimulated by electrical and magnetic physical factors, has emerged as a leading field of international academic research. Currently, numerous studies have focused on TES, TMS, and PES in the context of upper limb rehabilitation following a stroke. TES encompasses transcranial direct current stimulation (tDCS), a non-invasive technique that employs constant, low-intensity direct current to modulate the activity of cortical neurons. TMS, on the other hand, is a physical stimulation technique that utilizes a time-varying magnetic field to stimulate the cerebral cortex, generating induced currents that alter the action potential of cortical neurons, thereby influencing brain metabolism and neural electrical activity. Research has confirmed the efficacy of both tDCS and TMS in enhancing upper limb function post-stroke (37–42). In response to adverse reactions associated with TES, the development of high-precision electrical stimulation (HD-tES) and high-channel network electrical stimulation (Network-tES) has resulted in improved positioning accuracy and stimulation control (43–45). NMES and FES are techniques of peripheral stimulation. NMES employs low-frequency pulse currents to stimulate specific nerves or muscles, inducing muscle contraction and improving or restoring the autonomous movement of affected muscles or muscle groups, thereby promoting the recovery of upper limb function following a stroke. FES, a subset of NMES, focuses on achieving functional outcomes. Relevant studies have confirmed the efficacy of both techniques in enhancing upper limb function after stroke (46–48). A meta-analysis indicated that FES resulted in greater improvements in upper limb hemiplegia among stroke patients (49).

Intelligent rehabilitation refers to the application of computer technology and intelligent equipment to deliver personalized, precise, and efficient rehabilitation training for stroke patients, aiding them in restoring their functions and enhancing their quality of life. Currently, this approach encompasses virtual reality (VR) training, intelligent rehabilitation devices, and brain-computer interface (BCI) technology. VR training utilizes computer technology to simulate a three-dimensional environment, allowing patients to engage in interactive upper limb strength and exercise training through virtual reality equipment. This method aims to improve muscle strength and coordination of the upper limbs, thereby enhancing the athletic capabilities of the arms and hands (50–54). Intelligent rehabilitation devices can provide personalized rehabilitation training tailored to patients’ specific conditions and offer targeted feedback and adjustments through real-time monitoring of their movements. This approach yields significant direct benefits for upper limb motor control and functional activity in stroke patients (55). Currently, various robotic exoskeletons for upper limb rehabilitation have been developed, including upper limb exoskeletons (56), elbow exoskeletons (57), and forearm exoskeletons (58) that assist in extending upper limb joints such as the shoulders, elbows, and forearms. In addition, a range of hand and finger assistive devices has been created to enhance the fine motor skills of stroke patients’ hands. These include manipulators, gloves (59, 60), wrist exoskeletons (61), finger exoskeletons (62–64), thumb exoskeletons (65), and index finger exoskeletons (66), which collectively improve the grasping ability, stretching ability, and coordination of stroke survivors’ hands. The widespread application of intelligent rehabilitation techniques indicates that telerehabilitation and home-based rehabilitation have significant feasibility and potential for upper limb rehabilitation in stroke patients (67–69). By analyzing specific brain signal patterns, brain-computer interfaces (BCI) enable patients to interact with computers or machines, allowing them to perform exercise training through mind-controlled robotic arms or other assistive devices. This approach enhances upper limb function and improves muscle coordination and hand function in stroke patients (70–73).

#### 4.3.2 Assessment tools of post-stroke upper limb function

Currently, researchers are focusing on the functional evaluation of upper limb dysfunction after stroke, which is categorized into scale tools and intelligent assessment tools, and some small-scale pilot studies were carried out to verify its safety and feasibility.

Commonly used clinical scale tools include the Brunnstrom Rating Scale (BRS), the Fugl-Meyer Assessment (FMA), the Wolf Motor Function Test (WMFT), the Modified Ashworth scale (MAS), the Action Research Arm Test (ARAT), and the Box and Block Test (BBT). FMA is one of the most widely used scales in the quantitative assessment of motor function after stroke, which is further accurate and quantified by BRS. At present, several versions of FMA assessments have been carried out in Romania (74), Germany (75), Italy (76), South Korea (77), Italy (78), and other countries, confirming that FMA is an effective tool for measuring upper limb function after stroke with high reliability and validity. ARAT is a commonly used clinical assessment scale for assessing the upper limb function of stroke patients. It aims to evaluate the ability of patients to perform a series of functional movements, including grip strength, grasping, coordination, and fine movements, in daily life with high reliability (79). The FAM and ARAT were used as the core set of clinical recommendations for clinical practice and research by the CAULIN (27). Studies have shown that WMFT (80, 81), MAS (82), and BBT (83) have good reliability and validity in evaluating upper limb function.

The three-dimensional analysis system is a system that detects the trajectory of the human body and analyzes the trajectory of the action. It has the advantages of high precision, real-time monitoring, and data visualization. A study has shown that a three-dimensional (3D) motion capture system based on computer vision can detect changes in fine hand motor skills (84). The spectral arc length (SPARC) is obtained by three-dimensional kinematic measurements using an electromagnetic motion tracking system during the reach and grasp movements. Studies have shown a high correlation between the recovery of FM-UE and the smoothness recovery of SPARC reflection in patients with moderate to mild stroke in the early stage (85). The upper limb rehabilitation robot can evaluate the patient’s motor function, daily life activities, pain, fine movements, motor coordination, rehabilitation treatment effect, and other aspects (86–89). Both of them can provide objective data for clinical rehabilitation, but they have not been widely used and popularized due to their high cost, complex operation, limited use environment, and high time cost of data processing.

#### 4.3.3 Mechanisms of post-stroke upper limb dysfunction

Neurorehabilitation is a central focus in the field of limb rehabilitation following a stroke. Research into mechanisms based on neural plasticity, cortical reorganization, brain functional connectivity, and brain functional remodeling has emerged as a significant topic within the study of upper limb function post-stroke. Studies have indicated that various central and peripheral interventions, including Constraint-Induced Movement Therapy (CIMT), robotic exoskeleton training, and physical factor stimulation therapy, all facilitate the recovery of upper limb function after a stroke through mechanisms involving the cerebral cortex and neural plasticity (90–98).

### 4.4 Limitation

As far as we know, this is the first time to conduct a bibliometric analysis of the research on upper limb dysfunction after stroke. We conducted a quantitative analysis of a large number of pieces of literature in the field of upper limb dysfunction after stroke from 2004 to the end of 2023, which more intuitively reflects the research content and research hotspots in this field. The analysis results can predict future research trends, thus focusing and leading future research directions.

However, our research still has some limitations: this survey uses only one database, WoSCC, resulting in limited information retrieval and introducing bias. However, it is important to recognize that WoSCC is the main database used in bibliometrics research, and the data obtained from WoSCC can reasonably reflect the status of most publications within a specific topic to a certain extent. CiteSpace analysis software analyzes the keywords rather than the full text, so the overall detection of the article can be omitted, which needs to be further interpreted in combination with the actual clinical work. Due to the long time span of the included literature, the expression of keywords is inevitably inconsistent, similar, or diverse, resulting in a certain deviation in the analysis. There is no internationally accepted or specified parameter setting standard process in the software, which may cause deviations due to different algorithms.

## 5 Conclusion

Overall, our bibliometric analysis provides comprehensive information on publications related to upper-limb dysfunction after stroke. Our findings suggest that the field of upper limb dysfunction after stroke is booming and has sparked great interest in the global research community. The United States is the core research force in this field. Neurorehabilitation and Neural Repair is the most influential core journal in this field. Northwestern University and Catherine E. Lang are the top contributors and authors in this field, respectively. The main research interests are the reliability and validity verification of assessment tools, the development of intelligent assessment tools, and the clinical randomized trials of various interventions. Among them, the Fugl-Meyer assessment scale has attracted wide attention. Physical factor therapy, occupational therapy, and intelligent rehabilitation are popular intervention techniques for upper limb rehabilitation after stroke. Upper limb dysfunction after stroke focuses on neural rehabilitation and studies on the mechanisms of neural plasticity. Robot-assisted treatment and evaluation has been a research hotspot and frontier in this field in recent years and has great potential.

## Data Availability

The original contributions presented in the study are included in the article/Supplementary material, further inquiries can be directed to the corresponding authors.
