# Supplementary material for: Research trends and hotspots of post-stroke upper limb dysfunction: a bibliometric and visualization analysis
Source: Front Neurol. 2024 Oct 2;15:1449729. doi: 10.3389/fneur.2024.1449729 (PMC11479973; doi:10.3389/fneur.2024.1449729)
Supplement: Supplementary file 1 [file Data_Sheet_1.docx]

Supplementary Material 2

Research trends and hotspots of post-stroke upper limb rehabilitation：a bibliometric study and visualization analysis

**Qingqing Tang1†, Mengmeng Sun2†, Xin yue Yang3, Min He2, Ren Sa4, Kaiqiang Zhang1, Bing Zhu5*, Tie Li 1***

Department of Acupuncture and Tuina, Changchun University of Chinese Medicine, Changchun 130117, Jilin Province, China.

*** Correspondence：**

Bing Zhu [zhubing@mail.cintcm.ac.cn](mailto:zhubing@mail.cintcm.ac.cn)；Tie Li [litie@ccucm.edu.cn](mailto:litie@ccucm.edu.cn)

**Explanation of the exclusion criteria:** The excluded articles consisted of two parts, one part was the literature that was completely unrelated to post-stroke upper limb rehabilitation through reading the title and abstract (n=139). The other part is that in some literatures, research topic has some relationship with post-stroke upper limb function but post-stroke upper limb function is as the concomitant symptom or disease that need to be identified. In short, the research content only centre on the epidemiology (such as demographic factors, risk factor), mechanisms, diagnosis, pathophysiology, screening, prevention, management or treatment of other post-stroke upper limb rehabilitation relevant disease but not post-stroke upper limb rehabilitation, it include literatures: Post-stroke upper limb impairment is one of the major complications after stroke, many articles about stroke will mention the accompanying symptom of post-stroke upper limb impairment, but the content of the articles is still to discuss the epidemiology, mechanism, imaging, treatment and so on of stroke, and they do not cover post-stroke upper limb impairment, so this part of literature is excluded (n=256); The research content centre on limb disordre but the upper limb is not the focus or involves lower limb (publication number=221). Although post-stroke upper limb impairment is mentioned in the abstract, the stoke and upper limb is not the focus (n=304). At the same time, because post-stroke upper limb impairment emphasizes the causal relationship between stroke and limb impairment, these articles of which upper limb impairment due to other causes but not directly due to stroke also need to be excluded (n=232).
