# Supplementary material for: Research trends and hotspots of post-stroke upper limb dysfunction: a bibliometric and visualization analysis
Source: Front Neurol. 2024 Oct 2;15:1449729. doi: 10.3389/fneur.2024.1449729 (PMC11479973; doi:10.3389/fneur.2024.1449729)
Supplement: Supplementary file 2 [file Table_1.docx]

Supplementary Material 1

Research trends and hotspots of post-stroke upper limb rehabilitation：a bibliometric study and visualization analysis

**Qingqing Tang1†, Mengmeng Sun2†, Xin yue Yang3, Min He2, Ren Sa4, Kaiqiang Zhang1, Bing Zhu5*, Tie Li 1***

Department of Acupuncture and Tuina, Changchun University of Chinese Medicine, Changchun 130117, Jilin Province, China.

*** Correspondence：**

Bing Zhu [zhubing@mail.cintcm.ac.cn](mailto:zhubing@mail.cintcm.ac.cn)；Tie Li [litie@ccucm.edu.cn](mailto:litie@ccucm.edu.cn)

# Supplementary Tables

**Table S1 The search strategy**

| **The search strategy for Web of Science core collection** | |
| --- | --- |
| **Number** | **Search terms** |
| #1 | TS=((“post stroke upper limb function”) OR (“upper limb function after stroke”) OR (“Upper Extremity Paresis”) OR (“post stroke upper limb rehabilitation”) OR (“post stroke upper limb exercise therapy”)) |
|  | Stroke[MeSH Terms] were searched in Pubmed |
|  | cerebral hemorrhage[MeSH Terms] were searched in Pubmed |
|  | Brain Ischemia[MeSH Terms] were searched in Pubmed |
|  | cerebral infarction[MeSH Terms] were searched in Pubmed |
| #2 | TS=((“stroke”) OR (“Cerebrovascular Accident*”) OR (“CVA*”) OR (“Apoplexy, Cerebrovascular”) OR (“Vascular Accident*, Brain”) OR (“Cerebrovascular Stroke*”) OR (“Stroke*, Cerebrovascular”) OR (“Apoplexy”) OR (“Cerebral Stroke*”) OR (“Stroke*, Cerebral”) OR (“Stroke*, Acute”) OR (“Acute Stroke*”) OR(“Cerebrovascular Accident*, Acute”) OR (“Cerebrum Hemorrhage*”) OR (“Hemorrhage*, Cerebral Parenchymal”) OR (“Intracerebral Hemorrhage”) OR (“Hemorrhage*, Intracerebral”) OR (“Brain Hemorrhage, Cerebral”) OR (“Hemorrhage, Cerebral Brain”) OR (“Brain Ischemia*”) OR (“Ischemia, Brain”) OR (“Ischemic Encephalopathy*”) OR (“Encephalopathy, Ischemic”) OR (“Ischemia*, Cerebral”) OR (“Cerebral Infarction*”) OR (“Infarction*, Cerebral”) OR (“Cerebral Infarct*”) OR (“Infarct*, Cerebral”) OR (“Cerebral Infarction, Left Hemisphere”) OR (“Left Hemisphere, Infarction, Cerebral”) OR (“Infarction, Left Hemisphere, Cerebral”) OR (“Left Hemisphere, Cerebral Infarction”) OR (“Cerebral, Left Hemisphere, Infarction”) OR (“Infarction, Cerebral, Left Hemisphere”) OR (“Subcortical Infarction*”) OR (“Infarction*, Subcortical”) OR (“Posterior Choroidal Artery Infarction”) OR (“Anterior Choroidal Artery Infarction”) OR (“Cerebral Infarction, Right Hemisphere”) OR (“Right Hemisphere, Cerebral Infarction”) OR (“Infarction, Right Hemisphere, Cerebral”) OR (“Right Hemisphere, Infarction, Cerebral”) OR (“Cerebral, Right Hemisphere, Infarction”) OR (“Infarction, Cerebral, Right Hemisphere”)) |
|  | Hemiplegia[MeSH Terms] were searched in Pubmed |
|  | Paresis [MeSH Terms] were searched in Pubmed |
|  | Exercise Therapy[MeSH Terms] were searched in Pubmed |
| #3 | TS=((“Hemiplegia*”) OR (“Hemiplegia*, Transient”) OR (“Transient Hemiplegia*”) OR (“Monoplegia*”) OR (“Hemiplegia*, Post-Ictal”) OR (“Hemiplegia, Post Ictal”) OR (“Post-Ictal Hemiplegia*”) OR (“Hemiplegia*, Crossed”) OR (“Crossed Hemiplegia*”) OR (“Hemiplegia*, Flaccid”) OR (“Flaccid Hemiplegia*”) OR (“Hemiplegia*, Spastic”) OR (“Spastic Hemiplegia*”) OR (“Paresis*”) OR (“Muscular Paresis*”) OR (“Paresis*, Muscular”) OR (“Muscle Paresis*”) OR (“Paresis*, Muscle”) OR (“Monoparesis*”) OR (“Upper Extremity Paresis*”) OR (“Extremity Paresis*, Upper”) OR (“Paresis*, Upper Extremity”) OR (“Brachial Paresis*”) OR (“Paresis*, Brachial”) OR (“Hemiparesis*”) OR (“Remedial Exercise*”) OR (“Exercise*, Remedial”) OR (“Therapy*, Exercise”) OR (“Rehabilitation Exercise*”) OR (“Exercise*, Rehabilitation”) OR (“Endurance Training”) OR (“Motion Therapy, Continuous Passive”) OR (“Muscle Stretching Exercises”) OR (“Plyometric Exercise”) OR (“Resistance Training”) OR (“Neurological Rehabilitation”)) |
|  | Upper Extremity[MeSH Terms] were searched in Pubmed |
| #4 | TS=((“Extremities*, Upper”) OR (“Upper Extremity*”) OR (“Membrum superius”) OR (“Limb*, Upper”) OR (“Upper Limb*”) OR (“Arm”) OR (“Axilla”) OR (“Elbow”) OR (“Forearm”) OR (“Hand”) OR (“Fingers”) OR (“Metacarpus”) OR (“Shoulder”) OR (“Wrist”)) |
| #5 | #2 AND #3 AND #4 |
| #6 | #1 OR #5 |
